# Bevacizumab in ovarian cancer: A critical review of phase III studies

**DOI:** 10.18632/oncotarget.13310

**Published:** 2016-11-11

**Authors:** Luigi Rossi, Monica Verrico, Eleonora Zaccarelli, Anselmo Papa, Maria Colonna, Martina Strudel, Patrizia Vici, Vincenzo Bianco, Federica Tomao

**Affiliations:** ^1^ Department of Medico-Surgical Sciences and Biotechnologies, Sapienza University of Rome, Oncology Unit, ICOT, Latina, Italy; ^2^ Oncology Unit, Dono Svizzero Hospital, Formia, Italy; ^3^ Division of Medical Oncology 2, Regina Elena National Cancer Institute, Rome, Italy; ^4^ Department of Gynaecology and Obstetrics, Sapienza University of Rome, Policlinico Umberto I, Rome, Italy; ^5^ Division of Medical Oncology A, Sapienza University of Rome, Policlinico Umberto I, Rome, Italy

**Keywords:** ovarian cancer, bevacizumab, biological therapy, anti-angiogenic therapy, chemotherapy

## Abstract

Bevacizumab (BV) is a humanized monoclonal antibody targeting vascular endothelial growth factor and it is the first molecular-targeted agent to be used for the treatment of ovarian cancer (OC). Randomized Phase III trials evaluated the combination of BV plus standard chemotherapy for first-line treatment of advanced OC and for platinum-sensitive and platinum-resistant recurrent OC. These trials reported a statistically significant improvement in progression-free survival but not in overall survival. Furthermore, BV effectively improved the quality of life with regard to abdominal symptoms in recurrent OC patients. Bevacizumab is associated with adverse events such as hypertension, bleeding, thromboembolism, proteinuria, delayed wound healing, and gastrointestinal events. However, most of these events can be adequately managed. This review describes the latest evidence for BV treatment of OC and selection of patients for personalized treatment.

## INTRODUCTION

Ovarian cancer (OC) is one of the most debated oncologic disease in western and industrialized countries for different reasons. It is the second most common malignant gynecological disease, constantly showing an increase of incidence and prevalence; moreover among all the gynecological tumors it shows the main highest of mortality; despite recent important advances in early diagnosis it is usually detected at a very advanced stage; finally its prognosis remains unsatisfactory even today, with poor survival rates, despite the current availability of many active and emerging drugs (in Europe, in women diagnosed between 2000 and 2007, mean 5-years survival was about 37.6%) [1].

There is also great debate and confusion about who is the specialist appointed to manage the patient with OC; often this uncertainty implies that OC is frequently treated in centers not sufficiently skilled by a multidisciplinary point of view. The recognized standard treatment for OC is aggressive cytoreductive surgery (when it is possible), followed by a chemotherapeutic association of platinum and taxanes, according to different schedules [2–5].

Even if this first line chemotherapy is highly active, with ORR >75%, a large number of patients recur or experience cancer progression, finally dying for this disease (only 10-30% experience long term survival).

These patients are candidates for second-line chemotherapy, unfortunately with conflicting and poor results [6–12]. Nevertheless recurrent OC may be chemosensitive to platinum and therefore patients can be still treated selected with platinum-based schedules. Usually ORR and TFI (treatment-free interval) are directly proportional. On the other site, patients not responding to platinum chemotherapy (platinum refractory) and those presenting early relapse, within six months of treatment with platinum (platinum resistant), need further chemotherapy with non-cross resistant drugs. According to this concept, it is clear that new strategies are needed in order to improve clinical outcome in these poor prognosis patients.

More than 10 years ago, scientific evidences showed that paclitaxel seemed to be more effective when given weekly and that a part of this additional benefit may be due to an antiangiogenic effect [13]. Thus in the last years, in order to improve patients prognosis, a large number of trials were developed, investigating new targeted agents and specific molecular targets involved in the onset and progression of OC, including anti-angiogenesis agents. A number of key pathways are involved in the angiogenesis process. Some of these pathways seem to play a preeminent role: PDGF, VEGF, angiopoietin-Tie2 receptor and fibroblast growth factor (FGF) [14]. In this context a strong evidence has developed confirming that alterations and disruption of angiogenesis mechanisms are involved in the progression of OC, activating tumor growth, progression and metastatic spreading [15–16]. Already it has showed that increased angiogenesis, evaluated with increased tumor microvessel density (MVD), plays an unfavourable therapeutic effect since it is related with a reduced delivery and availability of chemotherapeutic drugs; this mechanism impaired clinical outcome in many tumors, including OC [15–18]. According to these data, in the last years an emerging scientific interest developed in order to investigate the role of angiogenesis inhibitors in OC patients [19–22]. Moreover, it is important to remember that anti-angiogenic drugs show a synergical effect with conventional chemotherapeutic agents and a different toxicity profile, due to their different mechanisms of action. In this context, since these targeted molecules are generally less toxic compared to traditional chemotherapeutic drugs, we must better explore their role in the management of OC patients, as single agents or in association with other chemotherapeutic or targeted drugs, in order to treat more patients with innovative therapeutic schedules.

But, unfortunately, there is still much to discover: since there are still conflicting and uncertain results in term of clinical outcome in patients receiving anti-angiogenesis therapy, it is useful to evaluate their effective impact in OC conducting phase III trials, evaluating safety, PFS and OS (see Tables 1 and 2) . BV is a MAb targeting VEGFR ligand and is currently the most investigated angiogenesis inhibitor in cancer patients. This targeted agent inhibits neoplastic vascularization, reducing the formation of new blood vessels, their number, density, diameter and permeability [23]. We can certainly say that with BV, the first angiogenesis inhibitor approved by FDA [24], the era of anti-angiogenesis therapy in clinical oncology was started. According to the promising results in other tumors, in the last years many studies have started investigating the role of BV in OC, as single agent or associated with other traditional drugs, showing an interesting and promising activity also in patients with recurrent disease [25–30]. Despite the availability of many new angiogenesis inhibitors explored in the therapy of OC, still today BV is the most investigated drug in this area, proving more and more to be a drug reference for this disease. To confirm this, we report that in USA Clinical Trial website there are at moment about 114 international registered trials investigating the use of BV in OC [31].

**Table 1:** Characteristics of 4 Randomized Controlled Trials

	GOG -0218	ICON7	OCEANS	AURELIA
Overall population Experimental arm 1 Experimental arm 2 Control arm	1873 623 625 625	1528 764 764	484 242 242	361 179 182
Age	<60 yr or 60-69 yr or >70 yr	<60 yr or 60-69 yr or >70 yr	<65 yr or ≥65 yr	<65 yr or ≥65 yr
GOG/ECOG PS	0-2	0-2	0-1	0-2
Stage/debulking	Stage III≤1 or Stage III>1 or Stage IV	Stage I-III≤1 or Stage I-III>1 or Stage IV and inoperable Stage III	Surgery at relapse or no surgery at relapse	NR
Treatment Line time to start CT platinum-free interval	First line post surgery NA	First line post surgery NA	Recurrent ovarian cancer NA 6-12 mos or >12 mos	Recurrent ovarian cancer NA 6-12 mos or >12 mos
Experimental arm treatment 1	Cycles 1-6: C AUC6, P 175 mg/mq, BV 15 mg/kg (starting in 2 cycle) q3w; Cycles 7-22: BV 15 mg/kg q3w	Cycles 1-6: C AUC5/6, P 175 mg/mq, BV 7.5 mg/kg (starting in 2 cycle) q3w; Cycles 7-18: BV 7.5 mg/kg q3w	C AUC4, Gem 1000 mg/mq, BV 15 mg/kg q3w	P 80 mg/mq days 1, 8, 15, 22 q4w, T 4 mg/mq days 1, 8, 15 q4w (or 1.25 mg/mq days 1-5 q3w), PLD 40 mg/mq day 1 q4w, BV 15 mg/kg q3w
Experimental arm treatment 2	Cycles 1-6: C AUC6, P 175 mg/mq, BV 15 mg/kg (starting in 2 cycle) q3w; Cycles 7-22; placebo q3w			
Control arm treatment	Cycles 1-6: C AUC 6, P 175 mg/mq, placebo (starting in 2 cycle) q3w; Cycles 7-22; placebo q3w	Cycles 1-6: C AUC5/6, P 175 mg/mq, placebo (starting in 2 cycle) q3w; Cycles 7-18; placebo q3w	C AUC4, GEM 1000 mg/mq, placebo q3w	P 80 mg/mq days 1, 8, 15, 22 q4w, T 4 mg/mq days 1, 8, 15 q4w (or 1.25 mg/mq days 1-5 q3w), PLD 40 mg/mq day 1 q4w
Primary endpoint	PFS	PFS	PFS	PFS
Secondary endpoints	OS, QoL	OS, ORR	OS, ORR, median duration of response	OS, ORR, QoL, safety
Median follow up	17.4 mos	48.9 mos	42 mos	13 mos

**Table 2:** Results of 4 Randomized Controlled Trials

RCTs	PFS (mos)	HR; 95% CI	*p*-value	OS (mos)	HR; 95% CI	*p*-value	ORR (%)	OR; 95% CI	*p*-value
GOG-0218									
TCP	11.2	0.908; 0.759-1.040	0.16	38.7	1.036; 0.827-1.297	0.76	NA	NA	NA
TC	10.3			39.3			NA		
TCBV	14.1	0.717; 0.625-0.824	<0.001	39.7	0.915; 0.727-1.15	0.45	NA	NA	NA
ICON7									
TC	17.4	0.87;	0.04	44.6	0.93	0.85	48		
TCBV	19.8	0.77-0.99		45.5	0.83-1.05		67		<0.001
AURELIA									
CT	3.4	0.48;	<0.001	13.3	0.85;	0.174	11.8	NA	0.001
BEV-CT	6.7	0.38-0.60		16.6	0.66-1.08		27.3	9.6-27	
OCEANS									
GC+PL	8.4	0.484;	<0.0001	32.9			57.4	NA	<0.0001
GC+BV	12.4	0.388-0.605		33.6	0.95	0.65	78.5		

Abbreviations: RCTs randomized controlled trials; PFS progression-free survival; OS overall survival; ORR objective response rate; HR hazard ratio; OR odds ratio; CI confident interval; mos months; NA no available; TCP paclitaxel + carboplatin + bevacizumab; TC paclitaxel + carboplatin + placebo; TCBV paclitaxel + carboplatin + bevacizumab → bevacizumab; CT chemotherapy; BEV-CT chemotherapy + bevacizumab → bevacizumab; GC+PL gemcitabine + carboplatin + placebo; GC+BV gemcitabine + carboplatin + bevacizumab → bevacizumab.

## BEVACIZUMAB IN FIRST LINE TREATMENT

In the landscape of front-line OC therapy, two important recent trials investigated the employ of BV to chemotherapy: the GOG218 trial and the ICON7 trial.

In 2011 the GOG Group reported data from the randomized phase III GOG218 trial. In this study 1873 women with III-IV stage tumors (epithelial OC or fallopian/peritoneal cancer )were enrolled . The patients were free of therapy after surgical approach [21].

Women were randomly assigned to three different arms in order to evaluate BV activity when associated with standard schedule. Treatment cycles were given every 21 days and all patients received six cycles of chemotherapy with taxol and carboplatin. The control therapy was standard chemotherapy with paclitaxel and carboplatin (TC) plus placebo, added in cycles 2 trough 22 (*n* = 625); the BV-starting (TCP) therapy was standard chemotherapy associated to BV (at dose of 15 mg/kg) added in cycles two trough six. The schedule was followed by placebo (n = 625); the BV-throughout (TCBV) therapy was standard chemotherapy plus BV added in cycles 2 trough 22 (*n* = 623). TC therapy and BV (or placebo) were administered every 21 days. Primary endpoint was median PFS (mPFS) and results were similar in TC and TCP groups (*p* value was 0.16 and HR 0.908); in the study the association of TC with BV showed a mPFS significantly longer than TC schedule (*p* value < 0.001 and HR of 0.717) [21]. The authors investigated median OS (mOS) and quality of life (QoL) as secondary endpoints. Regarding mOS the three treatment groups didn´t show significant differences.In comparison with the controls, the risk of death was estimated about 1.036 (*p* value = 0.76) in the TCP group and 0.915 (*p* = 0.45) in the TCBV one. A specific parameter Trial outcome index (TOI) was used in order to evaluate QoL analyzing the functional assessment of ovarian cancer treatment; during maintenance therapy no significant difference has been observed between the three groups. In TCP and TCBV groups, QOL before cycle 4 and cycle 7 was lower than that of the TC group [21].

The second trial we are going to analyze is ICON 7, a randomized open label study of phase III, that investigated on the clinical impact of BV in combination with chemotherapy [22].

This two arms trial enrolled 1,528 OC women with the following characteristics: clear cell G3 OC I-IIA staging (G3), stage IIB-IV OC, carcinoma of the fallopian tube or peritoneal cancer. Two randomized groups were selected: TC therapy was given every 21 days for a total of six cycles in a group; in the second group BV was added (at 7.5 mg/kg) and was given concomitantly for 5-6 cycles and continued for 12 additional cycles (TCBV). Primary endpoint was mPFS and it was significantly prolonged with BV (HR 0.81; *p* = 0.0041) [22].

Secondary endpoints were mOS, QoL and ORR. With 48.9 months of follow up, in contrast with data for progression free survival, no mOS benefit was reported in patients assigned to the TCBV schedule (HR 0.85; *p* = 0.11). A subsequent exploratory evaluation was able to detect a significant difference in OS in a subgroup of patients with disease at fourth stage, not surgical resectable tumor or sub-optimally resected disease (stage III) with residual tumor >1 cm (defined as poor prognosis) and treated with BV (*p* value = 0.03). In low-medium risk cases there were no significant differencebetween two groups in term of OS (*p* value = 0.20). Regarding QoL, TCBV group experiencedworse results (*p* value < 0.0001). About ORR, in the TC group 48% obtained complete or partial remission versus 67% in TCBV group (*p* < 0.001) [22].

## BEVACIZUMAB IN OVARIAN RELAPSED CANCER

In Aurelia trial, 361 patients progressing within 6 months after the end of ≥ 4 platinum based chemotherapy cycles were enrolled [20]. Patients treated with more than 2 previous regimens of chemotherapy or patients with refractory tumor were excluded, as well as women with previous history of gastrointestinal diseases (fistulas, gastrointestinal perforation, bowel obstruction, gastrointestinal abscesses, neoplastic recto-sigmoid or bowel involvement).

Patients received single-agent chemotherapy according to the following options: paclitaxel with a weekly schedule, liposomal adriamicin, every 4 weeks, 5 days of therapy with topotecan repeated every 3 weeks.Women were assignedto be treated only with chemotherapy or with bevacizumab (15 mg/kg every 3 weeks or 10 mg/kg every 2 weeks in topotecan treated patients).Treatment was discontinued in case of tumor progression or unacceptable toxicity.

In the study cross over to BV were possible in case of clear evidence of progression. Patients in the BV-CT arm were treated with standard therapy without BV at progression.

Primary objective of the study was reached, because a significant increase of mPFS (3.4 versus 6.7 months, *p* value < 0.001 and HR = 0.48) with the addition of BV. The mPFS benefit was observed in all subgroups evaluated. The improvement was remarkable in women with ascites. RECIST and/or GCIG CA-125 criteria were used as response criteria; in the CT group 12.6% of patients experienced an overall response rate; in the BV-CT group 30.9% (*p* < 0.001).

According to RECIST, in 287 patients, ORR was observed in 11.8% of chemotherapy group versus 27.3% for BV associated with chemotherapy (*p* value = 0.001); mOS was different in two groups: 13.3 months in CT group versus 16.6 months in BV-CT group (HR = 0.85, *p* value < 0.174), without statistically significant difference.

In the Oceans trial [19], 484 patients with platinum-sensitive recurrent OC were randomized to treatment with Carboplatin and Gemcitabine (GC) associated to BV (15 mg/kg) or PL (from 6 to 10 cycles). Patients continued the treatment until disease progression.

CA-125 elevation alone was not enough to establish progression, that could be determined by clinical progression. Regarding to the use of subsequent treatment after disease progression, 88% of patients in PL arm were treated and 84% in BV arm, including BV in 31% of the PL group and 15% of the BV group.

Patients pretreated with Bevacizumab or other VEGF inhibitors and those with previous gastrointestinal perforations and obstructions,, abscesses, fistulas, were excluded. In the BV arm the median PFS was better than in the PL arm (12.4 months versus 8.4 months, p-value: *p* < 0.0001, HR = 0.484,); the ORR was increased with the addition of BV (78.5% *v* 57.4%; *p* < 0.0001). In the analyses of all subgroups, including age, performance status, platinum-free interval and surgical cytoreductione for relapsed disease, BV demonstrated significantly improved mPFS. OS was similar between the two groups (GC+BV: 33.6 months; GC+Placebo: 32.9 months; *p* value: 0.65, HR = 0.95); it appeared consistent in the two groups (median follow-up was 58.2 months in the GCBV arm and 56.4 in GCP arm). Post progression survival (PPS) was 24 months; this could be linked to the four months difference occurred in mPFS but not in mOS. [19]

The randomized phase III trial GOG213, compared the activity of carboplatin, taxol and gemcitabine with or without Bevacizumab in patients with ovarian cancer, fallopian tube cancer and peritoneal carcinoma.

Eligible patients must have experienced clinical complete response to taxane-platinum chemotherapy and a minimum of 6 months of treatment-free period without clinical evidence of progression. Also women receiving maintenance targeted or endocrine therapy were eligible, but after at least 4 weeks since last infusion of biological therapy.

Patients were randomized to chemotherapy if they were not candidates for surgical cytoreduction.

Exclusion criteria were the following: more than one previous regimen of chemotherapy, secondary cytoreduction, need of parenteral nutrition; previous bowel perforation and/or obstruction, active bleeding or high risk of bleeding linked to other pathologies. Primary outcome were OS; secondary outcome were QoL, safety, molecular and/or biochemical profiles, PFS.

GOG213 accrued 674 women with recurring disease after frontline or maintenance therapy, including prior use of BV.In case of biochemical recurrence, surgical randomization was not permitted and chemotherapy schedule alone should have been considered.

The aim of the trial was selected in order to analyze activity of BV (at 15 mg/kg) associated to carboplatin and paclitaxel in a schedule of 6 cycles followed by BV maintenance therapy versus chemotherapy alone.

Interim data were presented during the international Meeting of the Society of Gynecologic Oncology in 2015 [32].

An OS in the BV arm of 42.2 months was reported *vs* 37.3 in the chemotherapy arm (HR: 0.83, *p* value:056). A median PFS of 13.8 ms was reported in BV group *vs* 10.4 in chemotherapy arm (HR: 0.61, *p* value < 0.0001). A risk reduction of progressive disease (39%) and death (17%), was registered.

The recruitment for the second aim of the study (investigating on secondary cytoreduction before chemotherapy) is ongoing. Primary endpoint was OS for both objectives; secondary endpoints were: PFS, QoL, safety and toxicity.

## BEVACIZUMAB-RELATED TOXICITY

Main adverse events (AEs) following BV infusion arebleeding, thromboembolism, delayed wound healing, proteinuria, hypertension, and gastrointestinal adverse events (Table 3).

**Table 3:** Adverse events

Adverse events	ICON 7		GOG 0218			OCEANS		AURELIA	
	TC	TCBV+	TCP	TCBV	TCBV+	GCP	GCBV+	CT	CTBV
Hypertension (>2)	< 1	6				0.4	17.4	1	7
Neutropenia	15	17	57.7	63.3	63.3	21.9	20.6	17	16
Febrile neutropenia	2	3							
Proteinuria	< 1	1	0.7	0.7	1.6	0.9	8.5	0	2
Venous tromboembolic event	2	4						4	3
Arterial tromboembolic events	1	3						0	2
GI perforation	< 1	1						0	2
Fistula/ abscess	1	1						0	1
Nn CNS bleeding			0.8	1.3	2.1	0.9	5.7		
Abdoninal pain (> 2)			41.6	41.5	47			5	7
Trombocytopenia	2	3						2	1

Abbreviations: TC chemotherapy; TC-BEV chemotherapy + bevacizumab → bevacizumab; TCP paclitaxel + carboplatin + bevacizumab; TCBV paclitaxel + carboplatin + bevacizumab → placebo; TCBV+ paclitaxel + carboplatin + bevacizumab bevacizumab; GC+P gemcitabine + carboplatin + placebo; GC+BV+ gemcitabine + carboplatin + bevacizumab → bevacizumab. TC paclitaxel + carboplatin + placebo; CT+BV chemotherapy + bevacizumab → bevacizumab

Data from the ICON7 study, showed that 56% of the women in the TC arm and 66% of TCBV arm reported AEs of grade 3 or higher [22]. Treatment with BV was associated with different AEs. A grade 1-2 mucocutaneous bleeding was observed in 36% of patients in BV arm versus 7% of those in the control group [33]. Eighteen percent of women treated with antiangiogenetic therapy experienced hypertension versus 2% of control group. In the TCBV group 7% of women showed grade 3-4 thromboembolic events versus 3% of patients in the TC group [22]. One percent of patients recruited in experimental group presented GIP *vs* < 1% in the control group. In extended follow-up of OS, one gastrointestinal fistula grade 3 and 3 grade 2 accidents (sarcoidosis, cardiac failure, and feet fractures) were also reported, all occurring in patients treated with BV [33]. In the TCBV arm, patients receiving BV with their first cycle of chemotherapy had abscesses, fistulas, or gastrointestinal perforations in similar percentage compared to those that did not receive BV at first cycle (3% and 4%, respectively) [22].

In GOG218 therapy was discontinued, due to AEs, in a more consistent rate of pts recruited in TCP arm (15%) and TCBV (17%) than in the TC arm (12 %). It´s remarkable that 76% of these AEs occurred during the chemotherapy period.

Hypertension occurred more frequently with BV than placebo; for this reason drug discontinuation was reported in only 2.4% of pts in the TCBV arm [21]. The three groups of treatment were similar regarding other AEs such as proteinuria of grade 3 or greater, GIP or fistula, neutropenia of grade 4 and febrile neutropenia, venous or arterial thrombosis, and wound disruption. [21]. Fatal AEs were reported in 1.0% of patients in the TC arm, in 1.6% in TCP arm, and in 2.3% in the TCBV arm. Only proteinuria, hypertension, pain were more frequent with extended treatment than with chemotherapy schedule, in TCBV arm [21].

In Aurelia trial, there was a higher frequency with the use of BV of grade ≥ 2 proteinuria and hypertension. [20]. A grade ≥2 of GIP was observed in 2.2% of patients treated with BV-CT. The same toxicity was not observed in pts treated with CT alone. In the BV-CT arm, in which there was a higher chemotherapy exposure, peripheral sensory neuropathy and hand-foot syndrome were observed more frequently.

In Oceans trial [19], the overall incidence of serious AEs ( ≥ grade 3) was more frequent in BV treated patients than in placebo group (24.9% versus 34.8%). Grade ≥ 3 hypertension (17.4% *vs* < 1%), venous thromboembolism (2.6 % *vs* 4.0 %) and proteinuria (8.5% *vs* < 1%) occurred more frequently in the BV group. No differences in neutropenia and febrile neutropenia rates were observed in both groups. None new safety concerns were observed. GIP occurred in two patients of BV group.

In the GOG213 [32], AEs were more common in BV-containing treatment, but within expected percentages. Infections (13% *vs* 6%) thromboembolism (4% *vs* 1%), hypertension (12% *vs* < 1%), GIP or fistula/abscess (15% *vs* 4%) and proteinuria (8% *vs* 0%) were the main AEs of severe grade (≥3) for the BV arm versus chemotherapy arm. Two deaths were reported in the patients treated with chemotherapy and 9 in the BV arm (7 possibly related to therapy).

At ASCO 2015 the authors reported intervention related Patient Reported Outcomes (PROs) [34].

Patients assigned to CT arm (298; 88%) or BV-CT arm (302; 90%) were evaluable in term of QoL. Compliance to therapy was 93% at baseline; it lowered to 88% after cycle 3,to 83% after cycle 6, to 82% after 6 months and 78% after 12 months. The FACT-O TOI scores were similar at baseline. No differences were shown in treatment side effects related to BV, with the exclusion of an impaired physical well-being occurring before cycle 3, worsened by treatment (*p* value: 0.028).In CPB group there was a decrease in physical activity (*p* value: 0.0156). Difference of 3.4 points (*p* value: 0.048) after cycle 3 and 6.8 points (*p* value:0.0004) after cycle 6.

## META ANALYSES OF BEVACIZUMAB EFFICACY AND SAFETY

Several meta analysis studies investigated the level of efficacy and safety of BV in treatment of OC, analyzing PFS, OS and toxicities in 4 randomized, multicenter, phased III trials.

The first meta-analysis of the four RCTs (4246 patients) was performed by Ye Q [35].

Bevacizumab significantly increased ORR, compared with chemotherapy alone (OR = 2.17) and was significantly beneficial in first-line therapy (OR = 1.90) and in patients with recurrent OC (OR = 2.77).

The addition of BV significantly improved PFS (HR = 0.69), and the subgroup analysis found a significant increase in PFS for both first-line (HR = 0.83) and second line therapy (HR = 0.48). No significant differences in term of OS were reported (HR = 0.934).

Patients receiving BV had a greater risk ( 2.7 ) of grade ≥ 2 GI adverse event, of grade ≥2 hypertension, (4.6) of grade ≥ 3 proteinuria (4.9) and of arterial thrombosis (2). There was no difference in the risk of significant venous thromboembolism.

In second meta-analysis by Mingyi Zhou [36], 3621 patients were considered: 1808 received BV plus standard chemotherapy and 1813 received chemotherapy alone.

Improved PFS was observed with chemotherapy plus BV as adjuvant therapy after initial surgical treatment (HR = 0.82; *p* value :0.000). A significant improving of PFS was also shown with the use of chemotherapy plus BV in patients with platinum-sensitive and platinum-resistant disease (HR = 0.48; *p* value : 0.000).

An increased OS was observed with the association of chemotherapy plus BV (HR: 0.87; *p* value: 0.026) for patients treated with surgery with recent diagnosis of OC.

OCEANS study showed similar results in OS between two arms and this endpoint was not achieved, until publication of this meta-analysis, in AURELIA.

The benefit of BV in term of PFS in newly diagnosed OC patients was stratified basing on various prognostic factors. Women presenting an high risk of progressive disease (stage III, macroscopic residual tumor>1 cm and stage IV) received clinical benefit from BV, as well as patients with a lower risk of progressive disease (I, II and III stage, macroscopic residual disease ≤ 1 cm) (high risk: HR:0.72; *p* value: 0.001; low risk: HR: 0.77 ; p value:0.001).

ICON7 trial showed no benefit from BV treatment for women with low risk for progressive disease. This result could be related to ICON7 recruitment: also patients withI-II stage were included in low risk subgroup.

The benefit of BV addition was observed only in patients younger than 70 ( < 60 yr: HR:0.77; *p* value: 0.001; 60-69 yr: HR: 0.76; *p* value: 0.003), while in patients aged 70 years or more no significant advantage was observed (HR: 0.74; *p* value: 0.067).

BV benefit was observed either in patients with a high tumor grading and low tumor grading (high grading: HR: 0.76; *p* value: 0.000; low grade: HR:0.71; *p* value: 0.007), but it is remarkable that ICON7 data showed no benefit in those patients expressing lower tumor grading.

Performance status 1-2 patients benefited from BV (HR: 0.68; *p* value: 0.000), while patients with PS 0 didn´t show significant advantage (HR: 0.87; *p* value: 0.103).

In newly diagnosed OC, BV was linked to higher risk for events such as bleeding (RR = 3.63; *p* = 0.000), ATE (RR = 2.29; *p* = 0.003), hypertension of grade ≥ 2 (RR = 4.90; *p* = 0.000), GIP (RR = 2.90; *p* = 0.003), and with proteinuria grade ≥3 (RR = 6.63; *p* = 0.000).

The results in term of PFS and OS in the two dose groups were very similar (16.5 ms with 7.5 mg/kg and 15.6 ms with 15 mg/kg; HR = 1.04 for PFS; HR = 1.15 for OS).

In the GOG218 trial patients treated with the dose of 15 mg/kg of BV experienced a worse toxicity profile than patients treated with 7.5 mg/kg of BV in ICON7 trial: hypertension ≥ 2 (*p* = 0.003), GIP ≥ 2 (*p* = 0.028), proteinuria ≥ 3 (*p* = 0.017). Patients treated with lower dose of BV showed a higher rate of ATE (*p* = 0.000), with wound-healing infections and complications (*p* = 0.001) than with the higher dose (15 mg/kg).

Four trials involving BV in OC were analyzed in a more recent meta-analysis [37], which confirmed that BV, in first line treatment, significantly extended PFS (HR = 0.82) and OS (HR = 0.86). In second-line BV extended PFS (HR = 0.48), but did not enhanced OS (HR = 0.93).

In another meta-analysis [38], Jun Li investigated on the effects of antiangiogenetic therapy in OC patients in terms of survival. This study was developed according to a pool of 12 clinical trials, including 4 phase III trials involving BV. Subgroup analyses were conducted, basing on the anti-angiogenesis agents, and they showed that administration of BV, considering together the results of first and second line treatment, was related with an increased PFS but not OS (HR: 0.90; *p* value:0.08).

With regard to toxicity, Ranpura [39] evaluated Phase II or III randomised controlled trials that compared BV in association with targeted therapy or chemotherapy versus placebo or best supportive care in association with targeted therapy or chemotherapy. Unfavourable outcome of interest was incidence of fatal AEs (FAEs).

The trials selected investigated patients with several solid tumors. Patients received 2.5mg/kg or 5mg/kg BV per week along with any of the following treatments: irinotecan, fluorouracil, leucovorin, oxaliplatin, capecitabine, paclitaxel, carboplatin, cisplatin, gemcitabine, docetaxel, pemetrexed, interferon alpha and erlotinib.

Subgroup analyses were undertaken by treatment dose, trial date, tumor type and chemotherapy regimens. Sixteen RCTs (10216 patients) were included in the review: four phase II trials and 12 phase III trials. Median length of follow-up varied significantly, ranging from 6.7 to 28 months. The overall incidence of FAEs with BV was 2.5%. Bevacizumab addition was related with a consistent safety problem, for the increased risk of FAEs compared to standard therapy alone, with an RR of 1.46 (*p* = 0.01).

No statistically significant differences,between patients receiving high- or low-dose BV (*p* = 0.16), in risk of FAEs were observed. Moreover the authors did not observed statistically significant variation in risk of FAEs by tumour type (*p* = 0.13). This association depended on chemotherapeutic agents employed (*p* = 0.045).

Taxanes or platinum agents were related to an increased occurrence of FAEs with BV (RR = 3.49; *p* = 0.006) but risk increase was observed when they were used with other agents (RR = 0.85). The most common ones were the following: neutropenia (12.2%), GIP (7.1%) hemorrhage (23.5%). There was no significant association with year of trial starting. Addition of BV to chemotherapy or biological therapy increased treatment-related mortality compared to standard treatment without BV.

Hongxin Huang [40] investigated on the role of BV in the risk of FAEs in patients with solid tumors. In NSCLC (RR = 1.88), pancreatic cancer (RR = 1.83), prostate cancer (RR = 3.34) and OC (RR = 2.35) a higher occurrence of FAEs was shown.

According to subgroup analyses,based on different doses of BV was performed, with no significant difference among dose schedules regarding risk of FAEs (*p* value: 0.90). Another evaluation according to subgroup analysis was stratified selecting the chemotherapeutic agents used in association with BV. Trials were divided into two arms: chemotherapy with platinum and chemotherapy without using platinum. RR of BV in association with platinum was 1.54 *vs* 1.15 for non-platinum.

All the trials were further divided into two arms: taxanes and chemotherapy without them. RR with BV associated with taxanes was 1.60 *vs* a value of 1.14 for non-taxanes. Significant higher risk of toxicity was observed with the association of BV and platinum/taxanes chemotherapy with a RR of 3.57 (*p* value: 0.005).

In conclusion, these meta-analyses suggested that in OC patients chemotherapy and BV regimen followed by BV significantly in frontline setting lead to improving of PFS and OS; in recurrent OC an increase in PFS was observed.

Furthermore, considering toxicity and price, these meta-analyses found that cost-effectiveness was better in patients treated with the lower dose of BEV (7.5 mg/kg) than the dose of 15 mg/kg in first line treatment.Toxicities were similar in all kind of tumors [41–46].

However, the occurrence of FAEs could be more frequently in OC, both for comparison with other types of cancer and for schedule of chemotherapy most frequently used.

## COST-EFFECTIVENESS EVALUATIONS

All oncologists claim a study with clinical advantages for women suffering from OC, considering the costs of treatment as a secondary problem.Unluckily, the financial coast of cancer care reached more than $90 billion annually [47]. Beyond this, the advantage of adding BV to standard OC treatment is not so clear, because it´s based on the PFS, that is an end point which depends on the measure adopted to evaluate the treatment's efficacy. Moreover, the cost-benefit analysis of new drugs and therapies must consider economical costs other than efficacy and tolerability. Therefore, it is more and more important to obtain cancer therapies not only efficacious, but also cost-effective (CE) [48–52]. Furthermore, the most investigated and standard measurement was the ICER system ( incremental cost-effectiveness ratio ) considered as the cost of the therapy for life-year saved (LYS) using the following equation: ICER = (costA - costB)/(efficacyA - efficacyB).

Regarding the GOG218 study, in a recent costs analysis, the estimation of the drugs costs were calculated by using medicare system reimbursement, including costs due to BV, chemotherapeutic drugs, supportive treatments, infusional expenses, and including the cost deriving from other kinds of clinical approach ( for example : surgical and medical care for GIP ). For all patients treated in GOG218 trial, considering the PFS and GIP at the baseline estimates, the ICER was $479,712 per progression-free LYS (PF-LYS) for PCBV and $401,088 per PF-LYS for PCBV-BV [53].

In their evaluation, Cohn et al, using the GOG218 data, tested differing BV prices to determine the ideal cost to reach cost effectiveness; anyhow, only at a price of 25% of the actual BV cost does the ICER dip below $100,000 per PF-LYS [54]. Therefore, the use of BV associated to chemotherapy in locally advanced and metastatic OC patients was not CE. Regarding GOG218, Hensley ML et al highlighted that the authors provide a simplified cost-effectiveness analysis because the analysis does not consider any indirect costs, except these for management of GIP. Moreover, it´s considerable that most of these analyses evaluate the costs of improvements in OS or quality-adjusted OS comparing results with a standard limit for what may be considered CE: $50,000 per LYS or, more recently, $100,000 per LYS [55]. Furthermore, the use of PFS as key parameter in CE analysis presumes that 3.8 months of progression-free time were a concrete benefit for patients. Anyhow, CA125 and periodic instrumental evaluation are employed because the large part of recurrent OC are found in absence of symptoms. Therefore, the advantage of 3.8 months in radiographically progression free does not mean that BV improve patient's QoL. Moreover, considering the absence of any improvement in OS by BV, the cost effectiveness of PCBV-BV is not reaching the limit of acceptability. Only if PFS with PCBV-BV would be 32.1 months did the ICER approach under the cutoff of $100,000 per PF-LYS [56].

Chan JK et al, conduced a CE analysis based on therapy regimens and outcome of ICON7 trial. In ICON7 study, the use of BV at lower-dose and shorter-duration could reduce the value of the cost numerator in the CE equation. They evaluated the ICER in high-risk OC patients with a demonstrated OS benefit (HR = 0.64, p>0.002) [21, 56].The limit of this analysis was the use of a model referring to data deriving from a single prospective study.Regarding toxicity, authors did not consider minor complications because of their similarity between the two treatment groups of the ICON7trial.Furthermore, this study did not consider costs of following therapies, additional expences for the patients, other costs for caregivers. Time lost and other derived financial efforts from other professional contributions were also considered. [19–21, 58]. Anyhow, the cost of treatment per cycle were calculated based on Medicare payment for administration of chemotherapy; and considering an 8-month advantage in OS, the ICER of BV was $167,771 per LYS [59]. Considering an option with a value acceptable for new therapeutic purposes (ICER = $200,000 per LYS), the ICER of BV is CE [60, 61].

Mehta DA et al in their three-state Markov analysis used the ICER as incremental cost for QALY score derived (Quality adjusted life year ), including QoL and median PFS. This cost method of analysis considers lower QoL to establish a decrease in effectiveness. Moreover, because the potential impact of a new emerging drug in medical oncology (Biosimilar BV) was also considered, a 30% price reduction with this agent was calculated. In GOG218 trial, BEV addition determined an ICER of approximately $2,420,691/QALY; whereas, in ICON7 trial, ICER resulted in a global cost of $225,515/QALY [62]. Considering different patient populations for both the clinical trials, the strategy is cost-ineffective in the universal pool of patients of GOG218 trial and in the high-risk subset of individuals of the ICON7 trial [22]. Furthermore, the main improvement with the use of BV was CE in patients with stage IV disease ( about $ 126,169 / QALY), Performance status 1 according to ECOG system ($116,575/QALY) and in the sub-group of women with suboptimal and large residual disease ( $ 122,822/QALY ), as experienced in ICON7 trial [63, 64]. In this model, GOG218 was analyzed including QoL, with the result of worse ICER in regimens containing BV compared with those without BV. In ICON7, it´s possible to suppose that the employ of BV at a reduced dose, could be more CE than that in GOG218; however, even considering only the therapy of high risk arm, use of BV did not show to be CE, with the evidence that the influence of BV costs is strong [62, 65].However, the efficacy of BV is proven by the better PFS seen in patients treated with PCBV-BV compared to PC,but the results could be influenced by a percentage of patients who didn´t respond.More factors to detect subgroups of responders patients could aid to reach the aim of demonstrate the effectiveness of a novel therapeutic like BV [66].

Moreover, a number of studies have shown better PFS results with consolidation chemotherapy after cytoreduction and use of adjuvant chemotherapy with CP for OC.A system was prepared considering the GOG trials n.178 and n.218. The group 1 was treated with six cycles of chemotherapy according to CP schedule. Group 2 was treated with six cycles of CP and followed by twelve cycles of P (CP-P). Group 3 was treated with one cycle of CP, five cycles of CPBV, and sixteen cycles of BV (CPBV-BV). Main evaluation parameters of outcome were : OS, PFS, costs, QoL utility values and complications. ICER value for CP-P schedule was about $13,402/QALY gained respect to CP schedule, whereas comparing CPBV-BV with CP, ICER value was about $326,530/QALY. Therefore CPBV-BV schedule appears more expensive with less efficacy than CP-P schedule., then CP-P seemed to be the best choice.On the other hand, CPBV-BV would be the best purpose if it was able to increase OS over CP-P schedule (6.1 years). According to this experience, BV maintenance led to worse results in terms of effectiveness compared to P consolidation, and it resulted more costly. However, its acceptance was due to not confirmed benefit in OS in patients treated with extended use of and therapy related severe neurotoxicity.

Lesnock JL et al, reported an OS of 58 months in optimal cytoreducted patients ( < 1cm ) and 35 months for patients with poor residual disease. The samecompromise was used for PFS evaluation, establishing for optimal patients and suboptimal patients the values of 24 and 14 months respectively. This model revealed that CPBV-BV is more expensive, with less efficacy, than CP-P. Considering these data, CPBV-BV would never become the CE in terms of PFS by 10. Regarding OS, CPBV-BV would be the best CE option if the OS would be improved of 6.1 years beyond baseline CP-P improvement. If the total costs of CPBV-BV reach a value below 37% of the total expenses, ICER score would lower below the value of $100,000 respect to the CP schedule, while the best option is still CP-P schedule.The total cost of CPBV-BV schedule might be less than 12% to gain the CE preference. Considering an ICER value about $13,402/QALY for twelve extended cycles of P, the CP-P schedule is better than CP followed by BV consolidation in terms of CE.In order to affirm the role of biological therapy in OC treatment, survival advantages might be supported by an improvement of CE balance. [67].

## CONCLUSIONS

This review could help the clinicians to receive the most updated evidences concerning the use of Bevacizumab in OC, suggesting future developments too in this emerging area of investigation. Main differences between the two studies in first line treatment of OC patients were the following: eligibility criteria, investigational arms, drug dosing and duration of maintenance, cross-over possibility.

Regarding eligibility criteria, while in GOG218 only III and IV staging patients were enrolled, in ICON7 also women with stage I-IIA (with clear cell histology and/or grading 3) and stage IIB have been included [21,22]. Furthermore, in GOG218 there were three arms of treatment, considering TC, TCP and TCBV arms; in ICON7 only TC and TCBV arms were considered because no placebo control was provided. Bevacizumabwas administered at different doses inclinicaltrials: 15 mg/kg (GOG218 trial) and 7.5 mg/kg (ICON7 trial). No differences in PFS and OS were observed between the two doses of BV; furthermore, 15 mg/kg dose was linked to a higher frequency of AEs (mainly proteinuria and GIP). In GOG218, there was no advantage in OS, probably for the possibility to use crossover strategy, but similar same result has been shown in ICON7, in which cross-over was not allowed; maybe 7.5 mg/kg could not represent a right dose to give survival advantage in first line.

The duration of maintenance was 15 months in GOG218 and 12 months in ICON7. It is noteworthy that, in both studies, women were treated with BV for the period determined in each studies and not until disease progression. Although no differences in OS have been shown in both studies, a subgroup analysis of these trials demonstrated an advantage in the high risk patients; in GOG218, IV stage patients receiving BV, showed better OS results (40.6 months in TCBV arm *vs* 32.8 in TCP arm, HR = 0.73); in ICON7 trial, high risk patients showed a median value of OS of 30.3 ms in TC arm and 39.7 ms in TCBV arm (HR = 0.64, *p* = 0.002).

However, the absolute mPFS improvement with the use in first-line of BV associated to chemotherapy and followed by extended treatment was 3.8 months (seeGOG218), and 1.5 months (ICON 7). Assessment of PFS was performed in GOG218 trial using both CA125 marker and radiologic evidence of progressive disease. Censoring CA125 elevations, the PFS difference increases to 6.0 months. According to ICON7 and GOG218 results, it appears that the PFS difference was smaller in the first study, probably due to the choice of using a lower dose (7.5 mg/kg) of BV ; nevertheless we must also consider that in ICON7 study the patients recruited had a better prognostic profile than in GOG218 study. The difference in PFS was not significant in recurrent OC, showing a value of 4 months being 4 months in platinum-sensitive relapsed OC (OCEANS trial) and 3.3 months in platinum-resistant OC (AURELIA trial) [19, 20].

AURELIA trial observed a statistically significant 3.3 month improvement in PFS, very similar to the value observed other different studies (ICON7, OCEANS and GOG218 studies), which confirmed an important role of the association of BV with chemotherapy in different groups of OC patients [19, 20]. Nevertheless, according to the results of other randomized trials, none benefit in terms of OS was observed (13.7 months) in BV arm, considering the median OS of approximately 12 months observed in patients with platinum-resistant tumor. Instead, the association of chemotherapy and BV in AURELIA trial has shown to improve significantly ORR from 11.8% to 27.3% (*p* = 0.001) (similar to the OCEANS trial).

AURELIA trial seem to confirm that there are strong evidences supporting the possibility to improve the clinical outcome in OC, justifying treatment of ovarian cancer with a prolonged use of BV, mostly in patients requiring a palliation without unacceptable toxicity.

Tomao et al [68] criticized the use of BV in conjunction with paclitaxel in Aurelia trial, because all patients received one or two lines of treatment with taxanes, and it would have been better to use another drug active in OC. Furthermore, they said that in several meta-analyses BV therapy, associated with paclitaxel/docetaxel, was characterized by higher toxicity than in association with other chemotherapeutic drugs and advised against the use of taxanes in association with BV in second- or third-line chemotherapy in platinum-resistant and recurrent OC patients.

Aurelia trial authors [69] replied that these observations are in contrast with results deriving from other experiences that suggest that paclitaxel is a highly active and well-tolerated regimen in a weekly schedule in relapsed OC patients, inducing significant RR in women with paclitaxel resistant disease given every 21 days; the authors also showed in the AURELIA's exploratory analyses, that paclitaxel adminestered with a weekly schedule was able to give a high RR and a long mPFS, compared with PLD or topotecan. No deaths were documented in the cohort of pts treated with BV associated to weekly paclitaxel schedule.

Occurrence of peripheral sensory neuropathy may occur more frequently after prolonged treatment with chemotherapy; this toxicity may also be increased in case of extended therapy with BV and weekly paclitaxel, when a prolonged and persistent PFS, require to continue the treatment until progression or toxicity.

In the study patients were stratified according to different chemotherapeutic schedules by without specific randomization. The study indicated that mPFS improved significantly associating BV to chemotherapy in all three different groups of treatment. HRs were 0.46 in the paclitaxel cohort (10.4 *v* 3.9 months), 0.57 in PLD cohort (5.4 *v* 3.5 months), and 0.32 in topotecan cohort (5.8 months *v* 2.1 months). A higher ORR was observed in patients treated when BV was associated to paclitaxel (53.3% *vs* 30.2%) and topotecan (17.0% *vs* 0.0%); a lower advantage was shown with the association of BV and PLD (13.7% *vs* 7.8%). No significant different results in mOS was found between treatment arms for PLD (13.7 months with BV-CT *vs* 14.1 with CT alone; HR = 0.91) and for topotecan (13.8 months *v* 13.3 months; HR = 1.09). Better clinical results in terms of OS were observed in patients treated with paclitaxel and BV (22.4 *v* 13.2 months; HR = 0.65). In all the three cohorts of treatment the rates of cross-over from chemotherapy to BV was similar (with paclitaxel: 38%; with PLD: 39%; with topotecan: 41%).

Different trials in metastatic breast cancer revealed that BV associated to weekly paclitaxel is an effective and safe schedule. Several studies confirm that this combination may be more efficient than the association with other drugs [70]; in the past some exploratory studies showed that the combination of BV and paclitaxel could enhance antiangiogenic effects of BV. Unfortunately, in the GOG-262 trial this attractive hypothesis was not investigated [71]; while in Aurelia trial the use and the role of paclitaxel administered with a weekly schedule remains an investigational therapeutic strategy [63, 72].

Important limitations of these studies are the following: randomization procedures, different prognostic factors among the patients recruited in different trials, disease related different profiles. Certainly, these factors and not homogeneous patient characteristics could have influenced chemotherapy selection in the different trials. However, according to results of the study, there is full evidence supporting favourable effects on PFS, ORR, and OS with the association of BV with weekly paclitaxel. There were no differences in OS between the two groups, but we must consider that in the study crossover to BV was accepted without restrictions from chemotherapy groups and occurred in a large number of patients (about 40%).Another critical point of the study is the following: a BV-alone arm was not available reducing the possibility to analyze BV activity in an off-chemotherapy arm.In 113 patients (31%) experiencing ascites, 9 (17%) in chemotherapy arm and one (2%) in BV-chemotherapy arm underwent paracentesis from beginning of therapy.Although no benefit in mPFS was observed in all the patients (with or without ascites at baseline), the results suggest that the use of BV improved control of ascites.

Patient-reported outcomes were analyzed using the EORTC QLQ-OV28, given to the patients at baseline, after two or three cycles and until tumor progression [65]. Analyses compared the proportion of patients experiencing ≥ 15% improvement after 8 or 9 week of treatment and evaluating abdominal and GI symptoms scores. This benefit was major in BV-CT than CT group (21.9% *v* 9.3%; *p* = 0.002).The reached benefit was 25.0% *vs* 13.0% in the paclitaxel group, 20.0% *vs* 8.8% in the topotecan group and 21.1% *vs* 6.8% in the PLD group. These results may underestimate the PRO improvement BV, in fact 1/3 of patients had symptoms at baseline evaluation

AURELIA must to be considered the first study, investigating the role of BV in OC, to demonstrate a statistically significant improvement in PROs.

GOG 218 trial was not able to confirm the Aurelia's results in terms of improvement in PROs and ICON7 demonstrated slightly worse PROs [73, 74]. In fact, in first line settings, most patients reported few disease-related symptoms at the beginning of chemotherapy.

Unfortunately, OCEANS trial did not investigated as end point the PROs evaluation.

Furthermore, it is possible that the open-label characteristics of study design could have determined this bias. Moreover patients enrolled in the BV-CT arm could have reported more favorable PROs, because of the knowledge of receiving an additional drug.

Another critical issue about PROs is that early-progressed patients were not regularly evaluated in order to analyze PROs after 8 or 9 week of treatment, resulting therefore in a larger number of patients not receiving regular PRO comparative analysis both in the control and experimental arms.As a consequence of this clinical bias, effective rates in PRO benefit between the arms treated with BV and chemotherapy and the arms treated only with chemotherapy gave decreased and not statistically significant values .

About Oceans trial, Tomao et al [75] observed that the improvement in PFS wasnot able to generate an advantage in terms of OS and QoL. The choose of select PFS as the only criterion useful to identify the value of novel therapeutic strategies is wrong. According to positive predictive CA-125 value and modifications in many patients before the radiologic response, the use of CA-125 detection could be a correct strategy to assess and identify disease progression in clinical trial investigating innovative therapeutic protocols in recurrent OC .

GCI (Gynecologic Cancer Intergroup) suggested novel parameters in order to evaluate disease progression (during therapy) and recurrence (after treatment), stressing and underlining the necessity of evaluating strategic data (CA-125, CA-125 alone, HE4, symptoms, only RECIST criteria) in order to optimally develop therapy in patients experiencing stable measurable disease but with a CA-125 constant rising [76].

Other authors (Aghajanian et Coll), did not use Ca125 as a predictive factor of response to therapy without including results suggestive for biomarker modifications. Patients received BV until progression or unacceptable toxicity; RECIST criteria, used alone as eligibility criteria of inclusion and as predictive factor of response to therapy could produce negative suggestions, prolonging in an inappropriate way an inefficient therapy, with expensive costs and little improvement by clinical point of view.

GOG 213 reached the average survival beyond 40 months in patients with recurrent disease.

Indeed, many open questions are emerging about the role of BV in OC and many doubts still deserve clear explanations. [77].

Should BV be used alone or associated with chemotherapy? Is use of BV as single-agentless toxic in all the patients? Is it extended and maintenance therapy an effective part of front-line treatment?

Regarding duration of BV, an extended therapy could be associated with an improved clinical benefit but, at same time, with higher costsand greater toxicity.

AURELIA and ICON7 were two important and attractive not placebo-controlled trials suggestive for potential bias in the evaluation of toxicity and activity. Moreover, in trials investigating the role of BV in recurrent disease patients received BV until toxicity or progression, whereas in the two first-line trials women were treated with BV for 15 and 12 months. This difference could explain the lower safety profile in relapsed patients. Open questions include also the potential alternative use of BV in integrated therapy for OC; intraperitoneal chemotherapy plays still an experimental role like as dose-dense chemotherapy; the optimal dose of BV and its role in patients with BRCA mutations that can be treated with olaparib, the role of BV in patients with platinum-sensitive OC and in competition with olaparib and other PARP inhibitors, with still conflicting data in progress. Patients with high-grade serous OC could receive a VEGF-directed therapy like a PARPi for treatment; different studies support the combination of VEGF inhibitors with PARPi [78]. In AURELIA and OCEANS trials, PFS and QoL (clearly improved in AURELIA) demonstrate the significant clinical benefit of adding BV to chemotherapy in relapsed disease.The OS improvement in stage IV patients enrolled in GOG218 and in patients with elevated risk of relapse in ICON7 trialare consistent with the probability that BV is able to increase the clinical outcome in patients with OC. We can absolutely conclude that adding BV to chemotherapy could increasetumor control in OC,both in first-line therapy and in case of relapse disease.

However, the results of studies including BV have not showed improvementin OS for eitherprimary or relapsed disease; considering the high cost associated with this treatment, it is necessary to select patients carefully.

Several phase II and III trials are currently ongoing to evaluate the role of BV in several settings of patients (Table 4 and Table 5).

**Table 4:** BEVACIZUMAB IN OVARIAN CANCER - Clinical Trials Ongoing

ClinicalTrials.gov Identifier	Responsible Party	Detailed Description	Regimen
NCT02354131	Nordic Society for Gynaecologic Oncology	Recurrent platinum-sensitive EOC, PPC or FTC	Arm-1: Nir Arm-2: BV → Nir Arm-3: BV-Nir combo
NCT01735071	Mario Negri Institute for Pharmacological Research	EOC pts at first recurrence occurred 6-12 months after the last platinum	Arm-1: BV+TrabArm-2: BV+Trab+Cb
NCT01847677	Grupo Español de Investigación en Cáncer de Ovario	Advanced EOC pts	- Preoperative treatment: Cb+P ± BV(4 cycles) - Surgery - Post-Operative treatment: Cb+P+BV (3 cycles) → BV until 15 months
NCT02022917	Chyong-Huey Lai, Chang Gung Memorial Hospital	Postoperative platinum-based CT plus adjuvant/maintenance BV after neoadjuvant CT → surgery in advanced EOC pts	BV (from post-op cycle 2) for at least 3 cycles (best to 6 cycles) → BV for 17 cycles
NCT00129727	Massachusetts General Hospital	CT naive EOC pts	Cb+P+BV (6-8 cycles) immediately post-operatively →BV
NCT01551745	Gradalis, Inc.	Recurrent/refractory EOC pts participating in Study CL-PTL 105	Adjuvant Bi-shRNAfurin and GMCSF augmented autologous tumor cell vaccine (FANG™)+BV
NCT00744718	Vejle Hospital	Platin resistant EOC pts	BV+Cb
NCT01146795	Jason D. Wright, Columbia University	Neoadjuvant therapy for EOC, FTC and PPC	Cb+P+BV (3 cycles) → surgical cytoreduction→ Cb+P+BV (6 cycles)
NCT01131039	Emory University	Platinum-resistant EOC, PPC or FTC pts	Gem+BV until progression
NCT00097019	Genentech, Inc.	Platinum resistant, advanced EOC or PPC pts subsequently progressed either during treatment with Dox or Top or within 3 months of discontinuing treatment with Dox or Top	BV
NCT01097746	M.D. Anderson Cancer Center	First-line treatment in EOC, PPC or FTC	Cb+P (cycles 1-6) + BV (cycles 2-6)
NCT00511992	University of Oklahoma	Stage II and III EOC, PPC, FTC pts	Cis+P (cycle 1-6)+BV (cycles 2-6)→ BV (12 cycles)
NCT01305213	National Cancer Institute, US	Recurrent or persistent EOC, PPC or FC pts	Arm-1: Fos+BV Arm-2: BV
NCT00126542	National Cancer Institute, US	Recurrent EOC, PPC or FTC	Erl+BV
NCT01031381	University of Pittsburgh	Recurrent EOC, PPC or FTC	RAD001+BV
NCT00588237	MSKCC	Frontline of Stage II-III EOC, PPC and FTC	P-Cis(IP) [6 cycles]→ BV
NCT01739218	Hoffmann-La Roche	Unresectable, stage IIIC/IV EOC, FTC and PPC	Cb+P+BV
NCT01652079	Massachusetts General Hospital	Recurrent platinum-resistant EOC and PPC	CRLX101 + BV

Reference: https://clinicaltrials.gov/; Abbreviations: Cb= carboplatin; P= paclitaxel; D= docetaxel; Gem= gemcitabine; BV= bevacizumab; Erl= erlotinib; Tem= temsirolimus; Ixa= ixabepilone; Eve= everolimus; Let= letrozole; Top= topotecan; Tam= tamoxifen; Rida= ridaforolimus; Nir= niraparib; Mel= melphalan; Rev= revlimid; Dox= doxil; Vel= Veliparib; Pem= Pemetrexed; Cis= cisplatin; IV= intravenous; IP= intraperitoneal; →= followed by; SD= stable disease; PD= disease progression; PPC= Primary Peritoneal Carcinoma; FTC= Fallopian Tube Cancer; Fos= fosbretabulin-tromethamine; MSKCC= Memorial Sloan Kettering Cancer Center;

**Table 5:** BEVACIZUMAB IN OVARIAN CANCER: Phase III Trial - Clinical Trials Ongoing

ClinicalTrials.gov Identifier	Responsible Party	Detailed Description	Regimen
NCT01802749	National Cancer Institute, Naples	Second-line treatment in platinum sensitive EOC pts after a BV/CT First-line	Arm-1: PLD+Cb±BV Arm-2: Gem+Cb±BV Arm-3: P+Cb±BV
NCT01837251	AGO Research GmbH	Platinum-sensitive recurrent EOC pts	Arm-1: BV→Gem+Cb (6 cycles)→BV Arm-2: BV→PLD+Cb (6 cycles)→BV
NCT01081262	National Cancer Institute, US	Stage II-IV, or recurrent stage I EOC or FTC pts	Arm-1: Cb+P (6 cycles) Arm-2: Oxa+Cape (6 cycles) Arm-3: Cb+P+BV (6 cycles)→ BV (12 cycles) Arm-4: Oxa+Cape (6 cycles)→ BV (12 cycles)
NCT01239732	Hoffmann-La Roche	Front-line treatment of pts with EOC, FTC or PPC	P (8 cycles)+BV (36 cycles)+Cb (4-8cycles)
NCT01462890	AGO Study Group	First-line treatment of EOC, FTC or PPC	Arm-1: CB+P+BV (6 cycles)→ BV (16 cycles) Arm-2: CB+P+BV (6 cycles)→ BV (38 cycles)
NCT00951496	National Cancer Institute, US	EOC, PP or FTC at stage II, III, or IV with either optimal or suboptimal residual disease	Arm-1: Cb+P [6 cycles]+BV(cycles 2-22) Arm-2: P+Cb(IP) [6 cycles]+BV (cycles 2-22) Arm-3: P-Cis(IP) [6 cycles]+BV (cycles 2-22)
NCT00565851	National Cancer Institute, US	Platinum-sensitive, recurrent EOC, PPC or FTC	Arm-1: Cb+D/P Arm-2: Cb+D/P+BV Arm-3: Cb+Gem Arm-4: Cb+Gem+BV

Reference: https://clinicaltrials.gov/; Abbreviations: Cb= carboplatin; P= paclitaxel; D= docetaxel; Gem= gemcitabine; BV= bevacizumab; Erl= erlotinib; Tem= temsirolimus; Ixa= ixabepilone; Eve= everolimus; Let= letrozole; Top= topotecan; Tam= tamoxifen; Rida= ridaforolimus; Nir= niraparib; Mel= melphalan; Rev= revlimid; Dox= doxil; Vel= Veliparib; Pem= Pemetrexed; Cis= cisplatin; IV= intravenous; IP= intraperitoneal; →= followed by; SD= stable disease; PD= disease progression; PPC= Primary Peritoneal Carcinoma; FTC= Fallopian Tube Cancer; Fos= fosbretabulin-tromethamine; MSKCC= Memorial Sloan Kettering Cancer Center;

Therefore, valid predictive biomarkers are an important issue to maximize the efficacy of BV in the treatment of OC [79, 80].
